# The Gender-Specific Relationship between Nutritional Status, Physical Activity and Functional Mobility in Irish Community-Dwelling Older Adults

**DOI:** 10.3390/ijerph18168427

**Published:** 2021-08-10

**Authors:** Maeve Lorraine O’Connell, Tara Coppinger, Seán Lacey, Tijana Arsenic, Aoife Louise McCarthy

**Affiliations:** 1 Department of Biological Sciences, Munster Technological University, Rossa Avenue, T12 P928 Cork, Ireland; tijana.arsenic@mycit.ie (T.A.); aoife.mccarthy@cit.ie (A.L.M.); 2 Department of Sport, Leisure and Childhood Studies, Munster Technological University, Rossa Avenue, T12 P928 Cork, Ireland; tara.coppinger@cit.ie; 3 Department of Mathematics, Munster Technological University, Rossa Avenue, T12 P928 Cork, Ireland; sean.lacey@cit.ie

**Keywords:** older adults, functional mobility, nutritional status, physical activity level

## Abstract

Research suggests that both nutrition and physical activity can protect mobility in older adults, but it is yet to be determined whether these relationships are affected by gender. Thus, we investigated the gender-specific relationship between nutritional status, physical activity level and functional mobility in Irish older adults. A cross-sectional study was undertaken in 176 community-dwelling older adults (73.6 ± 6.61 years) living in Cork, Ireland. Nutritional status was measured using the Mini Nutritional Assessment-Short Form (MNA-SF) and physical activity was assessed via the Physical Activity Scale for the Elderly (PASE). Functional mobility was measured using the Timed Up and Go (TUG) test. The gender-stratified relationship between variables was assessed using Pearson’s correlations and multiple linear regression. Partial correlations (*p* < 0.05) were observed for TUG with PASE score in both genders, and with MNA-SF score in females, only. Multiple regression showed that physical activity was a predictor of TUG in both genders (β = 0.257 for males, β = 0.209 for females, *p* < 0.05), while nutritional status was a predictor of TUG in females, only (β = −0.168, *p* = 0.030). Our results suggest that physical activity is associated with functional mobility in both genders, while the relationship between nutritional status and mobility may be specific to older females. These findings may be of interest for the design of functional preservation strategies.

## 1. Introduction

Declining fertility rates and increased length of life have resulted in significant growth in the global elderly population [1]. Currently, there are an estimated 727 million persons aged 65 years living worldwide, with this figure expected to more than double by the year 2050 [1]. In response to this transition, healthy ageing is considered a high priority area on global policy agendas [2]; and initiatives to promote health, ensure good quality of life and prevent the burden of illness in older age are encouraged.

Ageing often induces a chain of adverse physiological events, with the ultimate consequence being functional deterioration and mobility disability [3]. As mobility dictates the ability to perform daily life activities and live independently, strategies to delay functional decline and preserve mobility are imperative, and the implementation of such strategies is an active research topic [4]. Understanding factors associated with mobility decline is an important primary step in guiding intervention development.

Our understanding of risk factors for mobility impairment has progressed significantly in recent years, with evidence implicating factors such as chronic diseases, reduced cognition, and psychological factors as significant contributors [5]. Only modifiable risk factors can be targeted through intervention, however, two of which include: (i) physical activity [6], and (ii) nutrition [7], which may protect mobility through various physiological mechanisms. It is yet to be determined, however, if these relationships are affected by gender. Gender differences have been observed in the relationship between lifestyle factors and several ageing-associated disorders, such as cognitive decline [8] and sarcopenia [9]. Further, it was recently reported that, potentially due to physiological differences, the relationship between diet and frailty is stronger in female subjects, compared to males [10]. Considering the need to tailor functional preservation strategies, modifiable risk factors for mobility decline should be explored for older men and women, separately.

The Timed Up and Go (TUG) test is a simple tool for measuring functional mobility, lower extremity strength and balance [11]. Individuals are instructed to rise from a chair, walk a 10 ft distance, turn, walk back to the chair, and return to the sitting position. TUG has been employed as a reliable indicator of several health deficits in this age group, including frailty [12], sarcopenia [13] and falls risk [14]. Understanding factors associated with TUG performance is therefore valuable in informing intervention design, and in helping us to gauge who is at risk for a range of impairments.

This study aimed to investigate whether the relationship between nutritional status, physical activity, and functional mobility (measured by TUG) in older adults is gender-specific, in addition to estimating the prevalence of impaired mobility in a cohort of community-dwelling Cork elderly.

## 2. Materials and Methods

### 2.1. Study Population

A total of 204 participants were recruited to participate in this cross-sectional study. Participants were volunteers aged ≥65 years who responded to advertisements in local newsletters, radio stations, community centres, health clinics, churches and promotional sessions delivered to elderly community groups in Cork city and county, a region in southern Ireland. Participant recruitment took place from February to June 2019 inclusive. Inclusion criteria included age (≥65 years), community-dwelling, ability to walk 15 ft (with the use of a walking aid, if necessary) and informed consent. Those who received a mini-cog [15] score of <3 (*n* = 9), were unable to walk (*n* = 1) or did not complete the TUG test (*n* = 18) were excluded from the study, resulting in a study sample size of 176 participants (*n* = 96 female, *n* = 80 male, 73.6 ± 6.61 years) with complete sets of data for analysis. Study participants attended health screening sessions at community settings across Cork where their nutritional status, physical activity level and physical performance by TUG was measured, from March to July 2019 inclusive. Written informed consent was obtained from all participants prior to the commencement of the research. Ethical approval for this study was granted by the Cork Institute of Technology Research Ethics Committee (Cork, Ireland) in December 2018. Written informed consent was obtained from all participants prior to the commencement of the study.

### 2.2. Nutritional Status

Nutritional status was assessed using the Mini Nutritional Assessment—Short Form (MNA-SF) [16]. The MNA-SF is a 6-item nutrition screening tool specifically developed for elderly subjects and classifies individuals as malnourished (score of ≤7), at risk of malnutrition (score of 8–11) or of normal nutritional status (score of 12–14). It comprises measurement of body mass index (BMI) and questions on food intake, weight loss, mobility, and stress/disease. BMI was measured as weight (kg)/height (m)^2^. Weight (kg) was measured using a calibrated Tanita body composition analyser (model DC-360s, Tanita, Tokyo). Participants were asked to remove footwear and outer clothing prior to stepping on the scales. To allow for clothing weight, 1.2 kg and 0.8 kg were subtracted from the weight readings for males and females, respectively, as recommended by Whigham et al. [17]. Height (cm) was measured using a SECA portable stadiometer (model 213, SECA, Hanover, MD, USA) following the protocol outlined by the European Health Examination Survey (HES) [18].

### 2.3. Physical Activity Level

Physical activity was measured using the Physical Activity Scale for the Elderly (PASE), a five-minute self-reported questionnaire based on household, leisure and occupational activity [19]. PASE derives an overall score based on activity intensity, frequency and duration during the prior 7 days. The PASE questionnaire was the selected tool for this study as it is quick and inexpensive to use, has a strong record of validity in older populations [20,21] and has shown suitability for use in research studies investigating the association between physical activity, health, and physical function in older individuals, even where the sample size is small [20]. Further, a recent systematic review of physical activity questionnaires for older adults recommended PASE as the optimum self-assessment tool for total physical activity level measurement in this age group [22]. Minor adaptations were made to the questionnaire to improve the relevance of listed examples for an Irish audience. For example, moderate-intensity activities such as ballroom dancing, ice-skating and softball were replaced with brisk walking, cycling with light effort and dancing for leisure in the adapted version.

### 2.4. Timed Up and Go (TUG) Performance

The TUG was performed using the protocol outlined by the Center for Disease Control and Prevention [23]. Participants wore their regular footwear and used a walking aid if required. A chair with a straight back and fixed arms, and with the seat positioned 46 cm above the ground was used. A 10 ft distance was marked from the front of the chair and participants were instructed to, on the word “Go”; rise from the chair, walk to the mark placed 10 ft away (at normal walking pace), turn, walk back to the chair and return to the sitting position. Participants undertook one practice round before the assessment. A stopwatch commenced on the word “Go” and stopped when the participant’s back was against the back of the chair after returning to the sitting position. A cut-off time of >10 s was used to define impaired mobility [24].

### 2.5. Statistical Analyses

Statistical analyses were performed using RStudio 1.2.1335 (RStudio, PBC, Boston, MA, USA) for Windows. Descriptive statistics were used to describe the characteristics of the study group. Continuous variables are presented as means and standard deviations (SDs) and categorical variables are presented as numbers (n) and percentages (%). Independent *t*-tests and Mann–Whitney-U tests (depending on whether the data fitted a normal distribution or not) were used to describe differences in numerical variables between genders, while Pearson’s chi-squared test was used for categorical data. Correlations between TUG and MNA-SF score and PASE score were established by calculating unadjusted Pearson’s correlation coefficients, followed by partial correlation coefficients adjusted for age (years), BMI (kg/m^2^) and area of residence (urban/rural). To visualise the relationships, partial regression plots were created (with regression line and standard errors) for MNA-SF score and PASE score against TUG time in males and females, separately. Multiple linear regression was then used to investigate the relationship between nutritional status, physical activity level, demographic variables, and TUG performance for each gender. Due to the considerably larger sample size required for the detection of interaction effects [25], a separate regression model was built for each gender, as opposed to including gender interaction effects in one model [26,27]. Variance inflation factors (VIF) were calculated to assess for multi-collinearity (a VIF of < 4 was used as a cut-point for inclusion in the model) [28] and Durbin–Watson and Shapiro–Wilk tests were applied to the residuals to ensure assumptions of homoscedasticity and normally distributed were met, respectively. Due to the small number of participants classified as malnourished (*n* = 2), those who were malnourished and at risk of malnutrition (*n* = 36) were grouped together for multiple regression analysis. Independent variables entered were age (years), area of residence (urban/rural), BMI (kg/m^2^) PASE score and nutritional status (normal/malnourished or at risk). Unstandardised regression coefficients, B (with 95% confidence intervals), and standardised regression coefficients, β, were calculated for each predictor in the models. A significance level of 5% was used for the interpretation of all inferential statistics.

## 3. Results

The main characteristics of the study sample are shown in Table 1. A total of 96 (56.8%) participants were female and 117 (66.5%) were living in an urban area. The average TUG time was 9.5 ± 2.38 s, and this was significantly (*p* = 0.026) longer for females (9.9 ± 2.65 s) than males (9.0 ± 1.92 s). Overall, 30.1% (*n* = 53) of the study group had impaired mobility (TUG >10 s) [22], and although more prevalent in females than males (36.5% vs. 22.5%), this difference was not statistically significant (*p* = 0.065). A total of 1.1% (*n* = 2) and 19.3% (*n* = 34) of the study population were malnourished and at risk of malnutrition, respectively. Males were significantly more physically active than females (*p* = 0.008). No other statistically significant differences were found.

**Figure 1:**
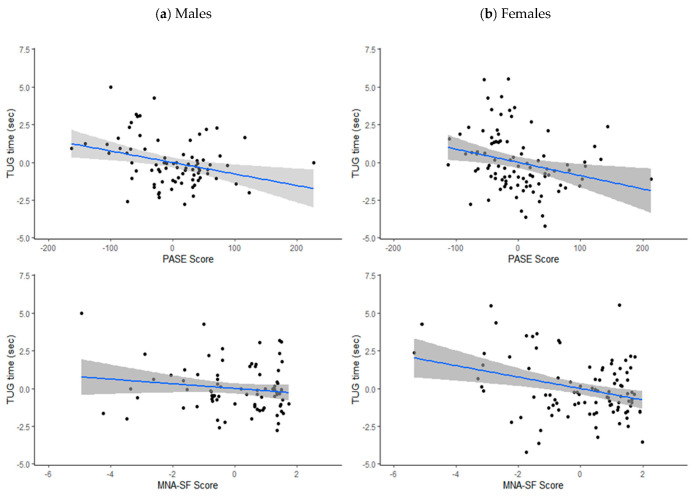
Partial correlation plots (adjusted for age, area of residence and body mass index) with standard error for PASE score and MNA-SF score with TUG time (s) in (**a**) males and (**b**) females.

Table 2 shows unadjusted and adjusted correlation coefficients for PASE score and MNA-SF score with TUG time, by gender, with partial correlation plots presented in Figure 1. Both prior to and after adjustment, PASE score was significantly correlated with TUG time in both genders (adjusted *p* = 0.006 for males, *p* = 0.011 for females), while MNA-SF score was significantly correlated with TUG score in females (*p* = 0.002), but not in males (*p* = 0.196).

No multi-collinearity was detected for any of the variables (all VIFs < 4) [28]. The predictors included in the multiple linear regression models (age, area of residence, PASE score, nutritional status, and BMI) explained a greater amount of the variation in TUG time for females, compared to males (Table 3, R^2^ = 37.4% for males, R^2^ = 49.9% for females). In both genders, increasing age was significantly associated with a longer TUG time (β = 0.499 for males, β = 0.533 for females; both *p* < 0.001), while higher PASE score was significantly associated with a shorter TUG time (β = −0.257 for males, β = −0.209 for females; *p* < 0.05). In females, a normal nutritional status (compared to malnourished or at risk) was significantly associated with a shorter TUG time (β = −0.168, *p* = 0.030). This relationship was not observed in males.

## 4. Discussion

The current study investigated the gender-specific relationship between nutritional status, physical activity and functional mobility as measured by the TUG in a cohort of Irish community-dwelling older adults. The prevalence of impaired mobility in this cohort was 30.1%, and was slightly, although not statistically significant, higher in females compared to males (36.5% vs. 22.5%). Although comparable data are limited, this figure is similar to the prevalence of slow gait speed (29.8%) reported from a recent analysis of older adults from six countries, including Ireland [29], and is slightly lower than the estimated prevalence of mobility limitations in US elderly (39.8%) [30]. No other data were found on the national prevalence of mobility impairment. However, the average TUG time in this cohort (9.5 ± 2.38 s) is in line with that previously reported for Irish older adults (9.0 ± 2.46 s) by The Irish Longitudinal Study of Ageing (TILDA) [31] and is almost identical to that estimated from a worldwide meta-analysis of twenty-one studies in older adults (9.4 s) [32]. Consistent with prior research in Irish older adults using the MNA [33], the prevalence of malnutrition in this group was low (1.1%).

Increasing age was associated with a longer TUG time in both genders, and the average TUG time was significantly longer for females, compared to males in this cohort (9.9 s vs. 9.0 s, *p* = 0.026), a finding that has also been reported elsewhere [34]. Males are taller than females, and stature is associated with gait speed [35]. However, it was recently suggested that gait speed may be affected by height in early ageing only and that this effect is attenuated as age increases beyond 65 years [36]. Another, perhaps more likely reason, may be the higher muscle mass and strength of males, compared to females [37]. This increased strength may translate to better TUG performance, as improved lower extremity strength is associated with better gait function [38]. Males also had significantly higher physical activity scores in this study (*p* = 0.008), a recurrent finding in this age group, as proposed by a systematic review of 53 studies [39].

Higher levels of physical activity are known to protect muscle mass by inducing alterations in skeletal muscle [40]. This can have a knock-on effect of also helping to protect bone mass [41], both important factors for mobility preservation in the ageing population. In the current study, self-reported physical activity level was significantly associated with TUG performance, irrespective of gender (β = −0.257 for males, β = −0.209 for females; *p* < 0.05). Research on the relationship between physical activity and physical function is generally indicative of a positive effect, and numerous interventions incorporating exercise programmes have led to improved functional outcomes in older adults [4]. Thus, a beneficial effect of physical activity on mobility was anticipated. However, it has been suggested elsewhere that physical activity level does not affect functional fitness when age is controlled for [42], while more recently, it was suggested that a relationship exists between physical activity and functional parameters in older males, but not females [9]. The reason for this conflict of evidence is unclear, but it may be due to the different outcome measures used in these studies, as opposed to TUG. For example, Rivera et al. [9] measured functional performance based on the 4-metre speed test, chair tests and handgrip strength, while Tuna et al. [42] included the 30-s chair-stand test, the 8-feet up and go test, and the 6-min walk test. The absence of a gold standard method for assessing performance is likely to introduce heterogeneity to results between studies.

Inadequate nutrition may contribute to impaired mobility due to factors such as feelings of exhaustion, weight loss and reduced activity and strength, and, indeed, research has shown that lower overall nutritional status is linked to an increased risk for disability [43]. In the current study, MNA-SF score was significantly correlated with TUG (partial r = −0.316, *p* = 0.002) and nutritional status was a significant predictor of functional mobility in older females (β = −0.168, *p* = 0.030). Interestingly, no such relationship was observed in older males, a finding that has not been reported elsewhere. However, gender differences have been reported in the association between nutrition and several health outcomes in adults, including frailty [10], obesity [27] and risk for hypertension [44], with stronger effects consistently reported in female subjects. Although exact mechanisms remain unclear, there are several physiological gender differences that may explain a more pronounced response to nutrition in females. Older females are at higher risk for chronic inflammation due to postmenopausal decreases in sex hormones [45], which may subsequently increase the risk for mobility decline through adverse effects on muscle [46]. In addition, this elevated risk may cause females to be more susceptible to chronic pain and depression and, consequentially, polypharmacy tends to be higher in females than males [47]. This could subsequently contribute to a stronger response to nutrition in females, as medication use is associated with a reduced level of physical activity [48], and central nervous system drugs such as opioids and anti-depressants increase the risk for mobility limitations [49]. Additionally, older females may be more sensitive to inadequate intakes of certain nutrients such as vitamin D and calcium, due to their elevated risk for bone loss [50], or inadequate protein intake due to the lower lean mass of older females, compared to males [51]. This may explain a greater impact of poor nutrition on mobility in females, as bone mass, muscle mass and functional parameters are strongly related [52]. It is also possible, however, that the relationship between nutritional status and functional mobility in males may have been attenuated by the relatively small (*n* = 176) sample size of the current study, and the fact that there were more female (*n* = 96) than male (*n* = 80) participants. Further research is needed to confirm these findings and to explore the underlying mechanisms which may explain gender differences in the relationship between nutritional status and mobility.

The limitations of this study must be considered when interpreting the findings. Firstly, the participants of this research were self-selected volunteers who responded to advertisements for the study in community settings. Therefore, those who are most likely to suffer from mobility impairment and malnutrition may not have been included, potentially resulting in a biased study sample that may not accurately represent the population. However, the average TUG time reported in this study is reassuringly comparable with that reported from a nationally representative study [31], indicating that such bias may have been marginal. Additionally, medication use by participants was not recorded in this study and was thus not controlled for in the analysis. This may somewhat limit the accuracy of the results observed as medication use can have an impact on both physical activity and functional mobility level [48,49]. Finally, cross-sectional analyses are unable to identify cause-effect relationships, and further investigation with a longitudinal study design would be useful in confirming the direction of the associations observed. However, the cross-sectional study design was suitable for the objectives of this study, which was exploratory in nature.

## 5. Conclusions

The results of this study suggest that a higher level of physical activity is associated with improved functional mobility in older adults, irrespective of gender. However, better nutritional status was associated with better functional mobility in female subjects, only, implying that the relationship between nutritional status and mobility may be gender-specific. Reasons for this gender difference warrant a more detailed, longitudinal investigation, in order to further define critical components for tailored functional preservation strategies for both genders.

## Figures and Tables

**Table 1:** Characteristics of study sample.

Characteristic	Males (*n* = 80)	Females (*n* = 96)	Total (*n* = 176)
Age; y, mean ± SD	73.8 ± 6.31	73.5 ± 6.87	73.6 ± 6.61
Living in urban area; *n* (%)	58 (72.5)	59 (61.5)	117 (66.5)
BMI; kg/m^2^, mean ± SD	28.5 ± 3.42	28.3 ± 5.56	28.4 ± 4.70
**TUG;** **s, mean ± SD**	9.0 ± 1.92 *	9.9 ± 2.65 *	9.5 ± 2.38
Impaired mobility: TUG ≥10 s; *n* (%)	18 (22.5)	35 (36.5)	53 (30.1)
**PASE Score; mean ± SD**	150.9 ± 65.74 *	131.5 ± 66.16 *	140.3 ± 66.50
**Nutritional status; *n* (%)**			
Normal status (score 12–14)	68 (85.0)	72 (75.0)	140 (79.5)
At risk of malnutrition (score 8–11)	12 (15.0)	22 (22.9)	34 (19.3)
Malnourished (score 0–7)	0 (0)	2 (2.1)	2 (1.1)

BMI body mass index; PASE physical activity scale for the elderly; TUG timed up and go; SD standard deviation; * *p* < 0.05 between genders (calculated by Mann–Whitney-U test).

**Table 2:** Pearson’s (r) and partial r † correlation coefficients of PASE score and MNA-SF score with TUG time for males and females (*n* = 176).

Variable	Males (*n* = 80)		Females (*n* = 96)	
	R	Partial r	R	Partial r
**PASE Score**	−0.349 **	−0.308 **	−0.454 ***	−0.262 *
**MNA-SF score**	−0.121	−0.149	−0.313 **	−0.316 **

PASE physical activity scale for the elderly; MNA-SF mini nutritional assessment-short form; † adjusted for age, body mass index and area of residence; * *p* < 0.05; ** *p* < 0.01; *** *p* < 0.001.

**Table 3:** Regression coefficients (B) and standardised regression coefficients (β) with 95% confidence intervals (95% CIs) for independent variables with TUG time in males (R^2^ = 37.4%) and females (R^2^ = 49.9%).

Independent Variables	B	95% CI (for B)	β	*p*-Value	VIF
**Males** **(*n* = 80)**					
Intercept	−3.909	-	-	-	-
**Age**	0.151	[0.096–0.207]	0.499	<0.001	1.06
BMI =	0.104	[−0.001–0.209]	0.186	0.051	1.11
Residence (rural)	0.472	[−0.327–1.271]	0.113	0.243	1.15
Nutritional status (normal)	−0.409	[−1.369–0.551]	−0.077	0.398	1.03
**PASE score**	−0.007	[−0.013–−0.002]	−0.257	0.008	1.11
**Females** **(*n* = 96)**					
Intercept	−4.962	-	-	-	-
**Age**	0.205	[0.142–0.269]	0.533	< 0.001	1.29
BMI	0.067	[−0.002–0.137]	0.141	0.058	1.03
Residence (rural)	0.509	[−0.317–1.334]	0.094	0.224	1.12
**Nutritional status (normal)**	−1.020	[−1.936–−0.104]	−0.168	0.030	1.09
**PASE score**	−0.009	[−0.015–−0.002]	−0.209	0.014	1.31

BMI body mass index; MNA-SF mini nutritional assessment-short form; PASE physical activity scale for the elderly; VIF variance inflation factor.

## Data Availability

The data presented in this study are available on request from the corresponding author.

## References

[1] United Nations World Population Prospects 2019 Highlights Report. https://population.un.org/wpp/Publications/Files/WPP2019_Highlights.pdf.

[2] World Health Organisation Ageing and Health. https://www.who.int/news-room/fact-sheets/detail/ageing-and-health.

[3] Navaratnarajah A., Jackson S. (2013). The physiology of ageing. Medicine.

[4] Di Lorito C., Long A., Byrne A., Harwood R.A., Gladman J.R.F., Schneider S., Logan P., Bosco A., van der Wardt V. (2021). Exercise interventions for older adults: A systematic review of meta-analyses. J. Sport Health Sci..

[5] Freiberger E., Sieber C.C., Kob R. (2020). Mobility in Older Community-Dwelling Persons: A Narrative Review. Front. Physiol..

[6] Tsai L.T., Portegijs E., Rantakokko M., Viljanen A., Saajanaho M., Eronen J., Pantanen T. (2015). Objective physical activity and life-space. Scand. J. Med. Sci. Sports.

[7] Milaneschi Y., Tanaka T., Ferrucci L. (2010). Nutritional determinants of mobility. Curr. Opin. Clin. Nutr. Metab. Care.

[8] Azad N., Al Bugami M., Loy-English I. (2007). Gender differences in dementia risk factors. Gend. Med..

[9] Rivera J., Fonseca-Sanchez M., Rodriguez P., Garcia J., Palma I., Aristi G., Flores-Luce A., Garcia L., Trujillo Y., Queipo G. (2016). Physical Activity Protects Men but Not Women for Sarcopenia Development. Gerontol. Geriatr. Med..

[10] Xu X., Inglis S., Parker D. (2021). Sex differences in dietary consumption and its association with frailty among middle-aged and older Australians: A 10-year longitudinal survey. BMC Geriat..

[11] Podsiadlo D., Richardson S. (1991). The timed “Up & Go”: A test of basic functional mobility for frail elderly persons. J. Am. Geriatr. Soc..

[12] Savva G.M., Donoghue O.A., Horgan F., O’Regan C., Cronin H., Kenny R.A., Rosa Camelier F.W., Camelier A.A. (2013). Using timed up-and-go to identify frail members of the older population. J. Gerontol. A Biol. Sci. Med. Sci..

[13] Martinez B.P., Gomes I.B., Oliveira C.S., Ramos I.R., Rocha M.D., Forgiarini Júnior L.A., Rosa Camelier F.W., Camelier A.A. (2015). Accuracy of the Timed Up and Go test for predicting sarcopenia in elderly hospitalized patients. Clinics.

[14] Shumway-Cook A., Brauer S., Woollacott M. (2000). Predicting the probability for falls in community-dwelling older adults using the Timed Up & Go Test. Phys. Ther..

[15] Steenland N.K., Auman C.M., Patel P.M., Bartell S.M., Goldstein F.C., Levey A.I., Lah J.J. (2008). Development of a rapid screening instrument for mild cognitive impairment and undiagnosed dementia. J. Alzheimers Dis..

[16] Rubenstein L.Z., Harker J.O., Salvà A., Guigoz Y., Vellas B. (2001). Screening for undernutrition in geriatric practice: Developing the short-form mini-nutritional assessment (MNA-SF). J. Gerontol. A Biol. Sci. Med. Sci..

[17] Whigham L., Schoeller D., Johnson L., Atkinson R. (2012). Effect of clothing weight on body weight. Int. J. Obes..

[18] FEHES Recommendations Measurement Protocols. https://ec.europa.eu/health/ph_information/dissemination/reporting/docs/fehes_protocols_en.pdf.

[19] Washburn R., Smith K., Jette A., Janney C. (1993). The physical activity scale for the elderly (PASE): Development and evaluation. J. Clin. Epidemiol..

[20] Washburn R.A., McAuley E., Katula J., Mihalko S.L., Boileau R.A. (1999). The Physical Activity Scale for the Elderly: Evidence for validity. J. Clin. Epidemiol..

[21] Dinger M.K., Oman R.F., Taylor E.L., Vesely S.K., Able J. (2004). Stability and convergent validity of the Physical Activity Scale for the Elderly (PASE). J. Sports Med. Phys. Fit..

[22] Sattler M.C., Jaunig J., Tösch C., Watson E.D., Mokkink L.B., Dietz P., van Poppel M.N. (2020). Current Evidence of Measurement Properties of Physical Activity Questionnaires for Older Adults: An Updated Systematic Review. Sports Med..

[23] Centers for Disease Control and Prevention Timed Up & Go. https://www.cdc.gov/steadi/pdf/TUG_test-print.pdf.

[24] Lee J.E., Chun H., Kim Y.S., Jung H.W., Jang I.Y., Cha H.M., Son K.Y., Cjo B., Kwon I.S., Yoon J.L. (2020). Association between Timed up and Go Test and Subsequent Functional Dependency. J. Korean Med. Sci..

[25] Leon A.C., Heo M. (2009). Sample sizes required to detect interactions between two binary fixed-effects in a mixed-effects linear regression model. Comput. Stat. Data Anal..

[26] Xiao Z., Xu H. (2020). Gender-Specific Body Composition Relationships between Adipose Tissue Distribution and Peak Bone Mineral Density in Young Chinese Adults. BioMed Res. Int..

[27] Zhao J., Sun J., Su C. (2020). Gender differences in the relationship between dietary energy and macronutrients intake and body weight outcomes in Chinese adults. Nutr. J..

[28] Hair J., Black W., Babin B., Anderson R. (2009). Multivariate Data Analysis.

[29] Greene B.R., McManus K., Redmond S.J., Caulfield B., Quinn C.C. (2019). Digital assessment of falls risk, frailty, and mobility impairment using wearable sensors. NPJ Digit. Med..

[30] Musich S., Wang S.S., Ruiz J., Hawkins K., Wicker E. (2018). The impact of mobility limitations on health outcomes among older adults. Geriatr. Nurs..

[31] Donoghue O., Ryan H., Duggan E., Finucane C., Savva G., Cronin H., Loughman J., Kenny R.A. (2013). Relationship between fear of falling and mobility varies with visual function among older adults. Geriat. Gerontol. Int..

[32] Bohannon R.W. (2006). Reference values for the timed up and go test: A descriptive meta-analysis. J. Geriatr. Phys. Ther..

[33] Power S.E., Jeffery I.B., Ross R.P., Stanton C., O’Toole P.W., O’Connor E.M., Fitzgerald G.F. (2014). Food and nutrient intake of Irish community-dwelling elderly subjects: Who is at nutritional risk?. J. Nutr. Health Aging.

[34] Ibrahim A., Singh D., Shahar S. (2017). ‘Timed Up and Go’ test: Age, gender and cognitive impairment stratified normative values of older adults. PLoS ONE.

[35] Bohanon R. (1997). Comfortable and maximum walking speed of adults aged 20–79 years: Reference values and determinants. Age Ageing.

[36] Elbaz A., Artaud F., Dugravot A., Tzourio C., Singh-Manoux A. (2018). The gait speed advantage of taller stature is lost with age. Sci Rep..

[37] Miller A.E.J., MacDougall J.D., Tarnopolsky M.A., Sale D.G. (1993). Gender differences in strength and muscle fiber characteristics. Eur. J. Appl. Physiol..

[38] Wu R., Zhang Y., Bai J.J., Sun J., Bao Z.J., Wang Z. (2020). Impact of lower limb muscle strength on walking function beyond aging and diabetes. J. Int. Med. Res..

[39] Sun F., Norman I.J., While A.E. (2013). Physical activity in older people: A systematic review. BMC Public Health.

[40] Cartee G.D., Hepple R.T., Bamman M.M., Zierath J.R. (2016). Exercise Promotes Healthy Aging of Skeletal Muscle. Cell Metab..

[41] Kim K.M., Lim S., Oh T.J., Moon J.H., Choi S.H., Lim J.Y., Kim K.W., Park K.S., Jang H.C. (2018). Longitudinal Changes in Muscle Mass and Strength, and Bone Mass in Older Adults: Gender-Specific Associations between Muscle and Bone Losses. J. Gerontol. A Biol. Sci. Med. Sci..

[42] Donat Tuna H., Ozcan Edeer A., Malkoc M., Aksakoglu G. (2009). Effect of age and physical activity level on functional fitness in older adults. Eur. Rev. Aging Phys. Act..

[43] Ge L., Yap C.W., Heng B.H. (2020). Association of Nutritional Status with Physical Function and Disability in Community-Dwelling Older Adults: A Longitudinal Data Analysis. J. Nutr. Gerontol. Geriatr..

[44] Song S., Kim J., Kim J. (2018). Gender Differences in the Association between Dietary Pattern and the Incidence of Hypertension in Middle-Aged and Older Adults. Nutrients.

[45] Abildgaard J., Tingstedt J., Zhao Y., Hartling H.J., Pedersen A.T., Lindegaard B., Dam Nielsen S. (2020). Increased systemic inflammation and altered distribution of T-cell subsets in postmenopausal women. PLoS ONE.

[46] Wang J., Leung K.S., Chow S.K., Cheung W.H. (2017). Inflammation and age-associated skeletal muscle deterioration (sarcopaenia). J. Orthop. Translat..

[47] Bijani A., Hasanjani Roshan A.R., Yazdanpour S., Hosseini S.R. (2014). Are older women likely to use medicines than older men? (Results from AHAP study). Caspian J. Intern. Med..

[48] Bertoldi A.D., Hallal P.C., Barros A.J. (2006). Physical activity and medicine use: Evidence from a population-based study. BMC Public Health.

[49] Boudreau R.M., Hanlon J.T., Roumani Y.F., Studenski S.A., Ruby C.M., Wright R.M., Hilmer S.N., Shorr R.I., Bauer D.C., Simonsick E.M. (2009). Central nervous system medication use and incident mobility limitation in community elders: The Health, Aging, and Body Composition study. Pharmacoepidemiol. Drug Saf..

[50] Alswat K.A. (2017). Gender Disparities in Osteoporosis. J. Clin. Med. Res..

[51] Schorr M., Dichtel L.E., Gerweck A.V., Valera R.D., Torriani M., Miller K.K., Bredella M.A. (2018). Sex differences in body composition and association with cardiometabolic risk. Biol. Sex. Differ..

[52] Shin H., Panton L.B., Dutton G.R., Ilich J.Z. (2011). Relationship of Physical Performance with Body Composition and Bone Mineral Density in Individuals over 60 Years of Age: A Systematic Review. J. Aging Res..

